# A Protocol for Multiple Gene Knockout in Mouse Small Intestinal Organoids Using a CRISPR-concatemer

**DOI:** 10.3791/55916

**Published:** 2017-07-12

**Authors:** Alessandra Merenda, Amanda Andersson-Rolf, Roxana C. Mustata, Taibo Li, Hyunki Kim, Bon-Kyoung Koo

**Affiliations:** ^1^Wellcome Trust - Medical Research Council Stem Cell Institute, University of Cambridge; ^2^Department of Genetics, University of Cambridge; ^3^Department of Pathology, Yonsei University College of Medicine

**Keywords:** Bioengineering, Issue 125, CRISPR/Cas9, knockout, organoid, intestine, electroporation, paralogues, Wnt.

## Abstract

CRISPR/Cas9 technology has greatly improved the feasibility and speed of loss-of-function studies that are essential in understanding gene function. In higher eukaryotes, paralogous genes can mask a potential phenotype by compensating the loss of a gene, thus limiting the information that can be obtained from genetic studies relying on single gene knockouts. We have developed a novel, rapid cloning method for guide RNA (gRNA) concatemers in order to create multi-gene knockouts following a single round of transfection in mouse small intestinal organoids. Our strategy allows for the concatemerization of up to four individual gRNAs into a single vector by performing a single Golden Gate shuffling reaction with annealed gRNA oligos and a pre-designed retroviral vector. This allows either the simultaneous knockout of up to four different genes, or increased knockout efficiency following the targeting of one gene by multiple gRNAs. In this protocol, we show in detail how to efficiently clone multiple gRNAs into the retroviral CRISPR-concatemer vector and how to achieve highly efficient electroporation in intestinal organoids. As an example, we show that simultaneous knockout of two pairs of genes encoding negative regulators of the Wnt signaling pathway (Axin1/2 and Rnf43/Znrf3) renders intestinal organoids resistant to the withdrawal of key growth factors.

**Figure:**
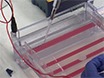


## Introduction

The reverse genetics approach is a widely used method for investigating the function of a gene. In particular, loss-of-function studies, in which disruption of a gene causes phenotypic alterations, play a key role in building our understanding of biological processes. The CRISPR/Cas9 method represents the most recent advancement in genome engineering technology and has revolutionized the current practice of genetics in cells and organisms. Cas9 is an RNA-guided endonuclease which binds to a specific DNA sequence complementary to the gRNA and generates a double-strand break (DSB). This DSB recruits DNA repair machinery that, in the absence of a DNA template for homologous recombination, will re-ligate the cut DNA strand via error-prone non-homologous end joining, which can thus result in insertions or deletions of nucleotide(s) causing frameshift mutations^1^.

The great ease and versatility of the CRISPR/Cas9 approach has made it a highly attractive tool for genome-scale knockout screens aimed at unraveling unknown gene functions^2^,^3^. Nevertheless, single gene knockout approaches are of limited use if multiple paralogues with redundant functions exist. Thus, ablating a single gene might not be enough to determine the function of that gene given possible compensation by paralogues resulting in little or no phenotypic alteration^4^. It is therefore important to knock out paralogues in parallel by delivering multiple gRNA vectors targeting the different paralogous genes in order to overcome the influence of genetic compensation.

To extend the use of CRISPR/Cas9 to paralogous gene knockout, we have recently developed a rapid, one-step cloning method to clone up to four pre-annealed gRNAs into a single retroviral vector^5^. The backbone, named CRISPR-concatemer, is based on an MSCV retroviral plasmid containing repetitive gRNA expression cassettes. Each cassette contains two inverted recognition sites of the Type IIS restriction enzyme *Bbs*I, which can be irreversibly replaced by an annealed gRNA oligo with matching overhangs using a Golden Gate shuffling reaction in a single tube^6^. This cloning method consists of repetitive cycles of digestion and ligation that allow simultaneous assembly of multiple DNA fragments by exploiting the different overhang sequences generated by *Bbs*I. The uniqueness of this enzyme is, for instance, the ability to perform asymmetric cuts outside of its recognition sequence; therefore, each cassette can have a different sequence with customized overhangs flanking *Bbs*I core site and in this way, each gRNA can be cloned in a specific position and orientation of the concatemer vector.

As a proof of principle, we demonstrated the use of this strategy in mouse intestinal organoids by disrupting simultaneously two pairs of paralogous negative regulators of the Wnt pathway by one round of electroporation^5^.

During the past few years, many other groups have developed similar strategies based on multiple gRNA expression vectors constructed using Golden Gate shuffling^7^ to achieve multi-gene knockout in various model systems, such as human cell lines^8^,^9^, zebrafish^10^ and *Escherichia coli*^11^. In their protocols, gRNAs are first cloned into individual intermediate vectors and then assembled together into one final product. By contrast, the main advantage of our CRISPR-concatemer strategy is the convenience of a single *Bbs*I shuffling, cloning step. Like other gRNA concatemers, our method makes possible either the simultaneous knockout of up to four different genes or increased CRISPR knockout efficiency following the targeting of one or two genes with multiple gRNAs (**Figure 1**).

In this protocol, we describe in detail every step in the generation of CRISPR-concatemer vectors, from gRNA design to the Golden Gate reaction and to confirmation of successful cloning. We also provide a highly efficient protocol for the transfection of CRISPR-concatemers into mouse small intestinal organoids by electroporation and subsequent growth factor withdrawal experiments.

## Protocol

### 1. gRNA Design for the CRISPR-concatemer Vector

Note: The aim of this section is to explain how to opt for the best targeting strategy and how to design gRNAs containing specific overhangs for the CRISPR-concatemer vector.

1. Design gRNAs against the genes of interest using a CRISPR gRNA design tool of choice. See the **Table of Materials** for an example. NOTE: When targeting a pair of paralogous genes, although it is possible to design one gRNA per gene, it is advisable to design two gRNAs per gene to increase the chances of achieving a double knockout (Figure 1).
2. Make sure the gRNAs do not contain the *Bbs*I recognition site by using a restriction mapping tool (see the **Table of Materials** for an example).
3. Add specific CRISPR-concatemer vector overhangs to each oligo, as shown in **Table 1**.

**Table:** 

	**Cassette 1**	**Cassette 2**	**Cassette 3**	**Cassette 4**
**Sequence (5′-3′)**	CACCGG[gRNA1]GT	ACCGG[gRNA2]G	CCGG[gRNA3]	ACACCGG[gRNA4]GTT
**Sequence (5′-3′)**	TAAAAC[RC-gRNA1]CC	AAAAC[RC-gRNA2]C	AAAC[RC-gRNA3]	CTAAAAC[RC-gRNA4]CCG

**Table 1: Overhangs for Each Cassette of the CRISPR-concatemer Vector.**

### 2. Cloning of gRNAs into the CRISPR-concatemer Vector

1. **Phosphorylation and annealing of oligos** NOTE: This step illustrates how to anneal top and bottom strands for each gRNA oligo and how to phosphorylate their ends in a single reaction.
  1. Prepare the reaction mixture for phosphorylating oligos and annealing top and bottom strands on ice, per the instructions below. NOTE: All oligos can be pooled into one reaction; for example, in the case of a 4 gRNA-concatemer vector, pool together 8 oligos.
  2. For 3 concatemers, use 3.0 µL gRNA top strand (1.0 µL from each gRNA; 10 µM, 1 µL/gRNA), 3.0 µL gRNA bottom strand (1.0 µL from each gRNA; 10 µM, 1 µL/gRNA), 2.0 µL T4 DNA ligase buffer (10x), 1.0 µL T4 PNK, and add H_2_O up to total volume of 20.0 µL.
  3. Mix well by pipetting and run this in a thermocycler using the following settings: 37 °C for 30 min, 95 °C for 5 min, ramp down to 25 °C at 0.3 °C/min, hold at 4 °C.
2. ***Bbs*I shuffling reaction** NOTE: In this section, the pre-annealed gRNA oligos are incorporated into the appropriate position of the concatemer vector in one step by alternating cycles of digestion and ligation.
  1. Dilute the reaction mixture 1:100 in DNase/RNase-free water to generate 3 and 4 gRNA-concatemer vectors. NOTE: When cloning 2 gRNA-concatemers this step is **not** needed.
  2. Assemble the *Bbs*I shuffling reaction on ice, per the instructions below. Include a negative control that only contains the vector.
  3. Use 100 ng CRISPR-concatemer vector, 10.0 µL oligo mixture, 1.0 µL BSA-containing restriction enzyme buffer (10x), 1.0 µL DTT (10 mM), 1.0 µL ATP (10 mM), 1.0 µL* Bbs*I, 1.0 µL T7 ligase, and H_2_O up to total volume of 20.0 µL.
  4. Mix well by pipetting and run this in a thermocycler using the following settings: Run **50 cycles** for cloning 3 and 4 gRNA-concatemers and **25 cycles** for 2 gRNA-concatemers, both at 37 °C for 5 min, 21 °C for 5 min, **hold at** 37 °C for 15 min , then 4 °C forever.
3. **Exonuclease treatment** NOTE: This step is highly recommended as it increases the efficiency of the cloning by removing any traces of linearized DNA.
  1. Treat the *Bbs*I shuffling reaction with a DNA exonuclease (see **Table of Materials**) as follows.
  2. Take 11.0 µL ligation mix from the previous step (2.2.3), add 1.5 µL exonuclease buffer (10x), 1.5 µL ATP (10 mM), 1.0 µL DNA exonuclease, and bring up total volume to 15.0 µL with water. Incubate at 37 °C for 30 min followed by 70 °C for 30 min. NOTE: This step removes any residual linearized DNA in the mixture and so increases the cloning efficiency.
  3. Use 2 µL of the reaction mixture for transformation into chemically competent *E. coli *bacteria by heat shock^12^. NOTE: Alternatively, the reaction can be stored for up to one week at -20 °C.
4. **Restriction digestion** NOTE: The aim of this step is to assess by restriction digestion the success of the cloning procedure.
  1. To confirm the presence of gRNA inserts in the CRISPR-concatemer vector, pick 4 - 8 bacterial colonies with an inoculating loop, grow each clone in 4 mL of LB medium overnight at 37 °C in an orbital shaker. Extract DNA using a plasmid miniprep kit according to the manufacturer's instructions (see the **Table of Materials**).
  2. Digest ~200 ng of DNA with 10 U* EcoR*I + 5 U *Bgl*II in a 10 μL reaction by incubating it at 37 °C for 3 h in a bacteria incubator. Include a separate reaction mixture with the corresponding original vector as a positive control for size comparison. NOTE: This will confirm whether all concatemers are present, since these two restriction enzymes will excise whole concatemers. These are the expected sizes for each concatemer: 2 gRNA-concatemer (800 bp), 3 gRNA-concatemer (1.2 kbp), and 4 gRNA-concatemer (1.6 kbp).
  3. Run digestion reactions on a 1% agarose gel at 90 V for approximately 20 min.
  4. Visualize the gel using a UV transilluminator. Identify the clones with correct insert size by ensuring their band pattern matches the one of the original vector and that each fragment has the expected size by using a DNA ladder.
  5. Digest the selected clones with 5 U *Bbs*I at 37 °C for 3 h in a bacteria incubator. Include a separate reaction mix with the corresponding original vector as a control. NOTE: This additional digestion step is to confirm that gRNAs have been cloned into the right position and consequently all *Bbs*I recognition sites have been lost.
  6. Run digestion reactions on a 1% agarose gel at 90 V for approximately 20 min. Consider as correct only the vectors that are not cut by *Bbs*I as they only contain gRNAs.
5. **Vector sequencing** NOTE: The aim of this step is to confirm the presence of gRNA sequences in those vectors identified as correct by restriction digestion analysis.
  1. Confirm positive concatemer vectors by Sanger sequencing^13^ using the following primers: Forward: TCAAGCCCTTTGTACACCCTAAG (for checking the first gRNA cassette) Linker1_Forward: GACTACAAGGACGACGATGACAA (for checking the second gRNA cassette) Linker2_Reverse: GGCGTAGTCGGGCACGTCGTAGGGGT (for checking the second gRNA cassette) Linker2_Forward: ACCCCTACGACGTGCCCGACTACGCC (for checking the third gRNA cassette) Linker3_Reverse: TCCTCCTCTGAGATCAGCTTCTGCAT (for checking the third gRNA cassette) Reverse: AGGTGGCGCGAAGGGGCCACCAAAG (for checking the last gRNA cassette)
  2. Check the presence of all gRNA by searching their sequences in the sequencing reads.

### 3. Transfection of Intestinal Organoids by Electroporation

NOTE: Please note that this procedure is based on the protocol published by Fujii *et al*. in 2015, with adaptation for mouse small intestinal organoid cultures^14^.

1. **Pre-electroporation** NOTE: This section describes how to prepare the mouse intestinal organoids prior to electroporation by removing all antibiotics and conditioned media from their culture medium. This will prevent possible toxic effects during electroporation.
  1. On day 0 of the transfection procedure, split organoids in a 1:2 ratio. NOTE: Intestinal organoid cultures can be obtained by performing crypt isolation according to previously established protocols^15^. Please refer to **Table 2** for all media compositions.
    1. When splitting organoids for electroporation, seed a minimum of 6 wells of a 48-well plate per transfection.
    2. Seed the organoids in 20 μL-basement matrix drops and grow them in WENR + Nic medium (Wnt + EGF + Noggin + R-spondin + Nicotinamide) at 37 °C, 5% CO_2_ in a humidified incubator (as previously described^15^).
  2. On day 2, change medium by replacing WENR+Nic with 250 µL of EN (EGF + Noggin) + CHIR99021 (Glycogen Synthase Kinase-3 inhibitor) + Y-27632 (ROCK inhibitor), without antibiotics (see **Table 2**). NOTE: In all the steps, the quantity of medium added to each well of a 48-well plate is 250 µL.
  3. On day 3, change the organoid medium to EN + CHIR99021 + Y-27632 + 1.25% v/v Dimethyl sulfoxide (DMSO), without antibiotics.
2. **Preparation of the cells** NOTE: Here we describe how to fragment organoids into small cell clusters by mechanical and chemical dissociation. These steps are critical to the success of the procedure.
  1. On day 4, disrupt the basement matrix domes using a 1 mL pipette tip and transfer organoids to a 1.5 mL tube. Pool contents of four wells of a 48-well plate into a tube.
  2. Mechanically break organoids into small fragments by pipetting up and down with a P200 pipette approximately 200 times. Centrifuge at room temperature, 5 min at 600 x g.
  3. Remove the medium and resuspend the pellet in 1 mL of a cell culture grade recombinant protease (see table of materials). Incubate at 37 °C for a maximum of 5 min and then check a 50-µL drop of sample under an inverted light microscope with a 4x objective. NOTE: Clusters of 10 - 15 cells are desirable, as this increases cell survival after electroporation.
  4. Transfer the cell suspension to a low-binding 15 mL tube and halt the dissociation by adding 9 mL of basal medium without antibiotics (see **Table 2**). Centrifuge at room temperature, 5 min at 600 x g, then discard the supernatant and resuspend the pellet in 1 mL of reduced serum medium (see **Table of Materials**).
  5. Count the number of cells with a Bürker's chamber and use a minimum of 1 x 10^5^ cells per electroporation reaction. Add 9 mL reduced serum medium to the 15 mL tube and centrifuge at room temperature, 3 min at 400 x g.
3. **Electroporation** NOTE: The following sections provide instructions on how to perform electroporation and to make organoids recover afterwards.
  1. Remove all of the supernatant and resuspend the pellet in an electroporation solution (see **Table of Materials**). Add a total amount of 10 µg DNA to the cell suspension and add electroporation solution to a final volume of 100 µL and keep the cell-DNA mixture on ice. Use CRISPR-concatamer vectors in combination with a Cas9 expression plasmid (*e.g.* Addgene #41815) in a 1:1 ratio. NOTE: The total volume of the DNA added should be less than or equal to 10% of the total reaction volume.
  2. Include a separate transfection mix containing a GFP plasmid to evaluate transfection efficiency (*e.g.* pCMV-GFP, Addgene #11153, or any generic GFP-expressing plasmid).
  3. Add the cell-DNA mixture to the electroporation cuvette and place it in the electroporator chamber. Measure the impedance by pushing the appropriate button on the electroporator and ensure that it is 0.030-0.055 Ω. Perform electroporation according to the settings shown in **Table 3**. NOTE: If the impedance value falls outside of the allowed range, adjust the solution volume in the cuvette.
  4. Add 400 µL of electroporation buffer + Y-27632 to the cuvette and then transfer all to a 1.5 mL tube. Incubate at room temperature for 30 min to allow cells to recover and subsequently spin them at room temperature for 3 min at 400 x g.
  5. Remove the supernatant and resuspend the pellet in 20 μL/well of basement matrix. Seed approximately 1 x 10^4^ to 1 x 10^5^ cells per well in a 48-well plate and add EN + CHIR99021 + Y-27632 + 1.25% v/v DMSO medium. Incubate at 37 °C.
  6. On day 5, change the medium to EN + CHIR99021 + Y-27632, and check transfection efficiency by observing GFP expression (Figure 2). Keep organoids at 37 °C and refresh EN + CHIR99021 + Y-27632 medium after 2 days.
  7. On day 9, change the medium to WENR + Nic + Y-27632 and incubate at 37 °C. NOTE: Y-27632 can be removed after 7-10 days (on day 16-19).

**Table:** 

	**Poring pulse**	**Transfer pulse**
**Voltage**	175V	20V
**Pulse length**	5 msec	50msec
**Pulse interval**	50msec	50msec
**Number of pulses**	2	5
**Decay rate**	10%	40%
**Polarity**	+	+/-

**Table 3: Electroporation Settings.**

### 4. Growth Factor Withdrawal

Note: Here it is exemplified how to conduct a growth factor withdrawal experiment when knocking out negative regulators of the Wnt pathway in intestinal organoids.

1. 10-14 days after electroporation, split the organoids in a 1:3 ratio in a 48-well plate following the above-mentioned steps (3.2.1 - 3.2.2).
2. Resuspend the organoid pellet in 20 µL of basement membrane matrix and let it solidify at 37 °C for 10 min. Then, overlay 250 µL of growth factor-deprived medium (*e.g.* EN) to test whether knockout of the target genes has been achieved^5^.
3. Split the organoids under growth factor-deprived conditions for a minimum of 2 - 3 passages to see a difference in survival between wild type wildtype (WT) control organoids and mutant organoids^5^,^15^. NOTE: Wildtype organoids should not be able to survive in growth factor-deprived medium over two passages, while mutant lines should be able to grow.

## Representative Results

In order to confirm the presence of the correct number of gRNA inserts in the concatemer vector, restriction digestion is performed with enzymes (*EcoR*I + *Bgl*II) flanking all gRNA expressing cassettes (each cassette size is ~400 bp, Figure 1). For example, when generating a 4 gRNA- concatemer vector, the expected size of the lower band in the agarose gel is approximately 1.6 Kbp; any band lower than this indicates that not all of the 4 gRNA cassettes are inserted into the vector (Figure 2A). In addition, it is always recommended to check that all *Bbs*I recognition sites are lost and the enzyme does not cut the vector (Figure 2B).

Once the constructs have been confirmed, they can be delivered to mouse intestinal organoids by electroporation to achieve optimal levels of transfection efficiency (up to 70%), as shown by the GFP control (Figure 3).

Finally, to functionally test the efficiency of this strategy, intestinal organoids transfected with Cas9 and concatemer vectors against Axin1/2 and Rnf43/Znrf3 were cultured in EN (R-spondin withdrawal) and EN + IWP2 (R-spondin and Wnt withdrawal, IWP2: Porcupine inhibitor, 2.5 μM) media for a minimum of 3 passages (Figure 4). While untransfected WT organoids died under both conditions, Axin1/2 knockout organoids survived in both due to downstream activation of the Wnt pathway; in addition, Rnf43/Znrf3 mutant organoids survive in the absence of R-spondin but cannot survive in the presence of IWP2, which causes depletion of the Wnt that activates the pathway. Taken together, these observations demonstrate that knockout of these pairs of paralogues is possible by generating the expected organoid phenotype. Details of these results have been published in *Developmental Biology*^5^.

**Figure:**
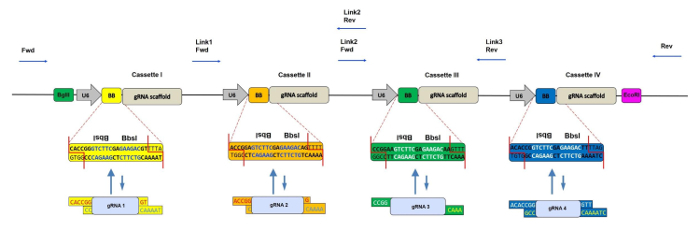

**Figure 1: Schematic Representation of the CRISPR-concatemer with 4 Cassettes. **Scheme of the 4 gRNA-concatemer vector with each 400 bp cassette containing a U6 promoter, two inverted repeated *Bbs*I sites (also indicated as BB) and gRNA scaffold in this order. During the shuffling reaction, *Bbs*I sites are replaced by gRNA fragments with matching overhangs and consequently lost. Binding sites of the sequencing primers for checking the correct insertion of gRNA oligos are shown by the blue arrows. Fwd = forward primer, Rev = reverse primer, Link 1/2/3 = linker regions 1/2/3. Please click here to view a larger version of this figure.

**Figure:**
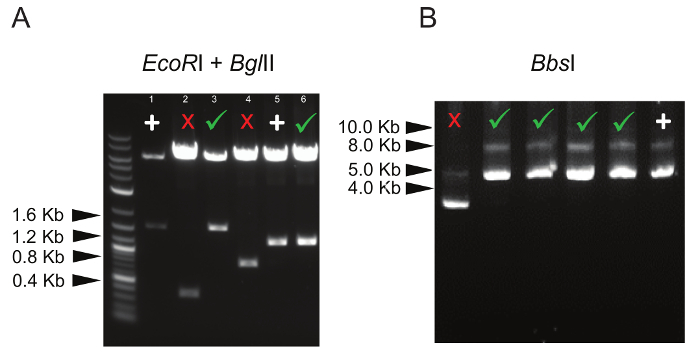

**Figure 2: Representative Digestion Patterns of Concatemer Vectors. **(**A**) Double digestion of 3 and 4 gRNA-concatemer vectors with *EcoR*I and *Bgl*II. The correct digestion pattern is marked by a green tick, whereas vectors with only 1 or 2 gRNA insertions are marked by a red cross. Lane 1 shows digestion of a 4 gRNA-concatemer parental vector used as positive control (marked by "+"); similarly, lane 5 shows digestion of a 3 gRNA-concatemer parental vector, marked by "+". (**B**) Digestion with *Bbs*I, showing the correct size of undigested concatemer vectors (indicated by the green ticks). Digestion of a gRNA-containing concatemer vector that has lost *Bbs*I sites is used as a positive control and is marked by "+". Please click here to view a larger version of this figure.

**Figure:**
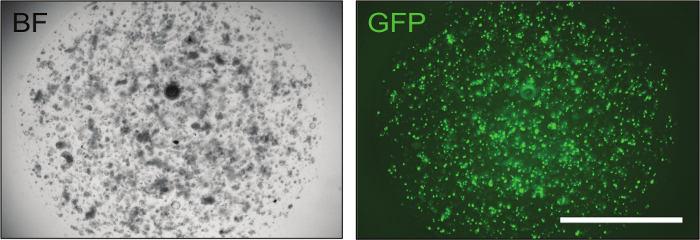

**Figure 3: Representative Image of Successfully Electroporated Intestinal Organoids. **Transfection of a GFP plasmid is instrumental to evaluate transfection efficiency. Approximately 24 h after electroporation, organoids containing a small number of cells are already visible and, if the electroporation procedure was successful, up to 70% of them displays green fluorescence. BF = bright field, GFP = green fluorescent protein. Scale bar = 2,000 µm. Please click here to view a larger version of this figure.

**Figure:**
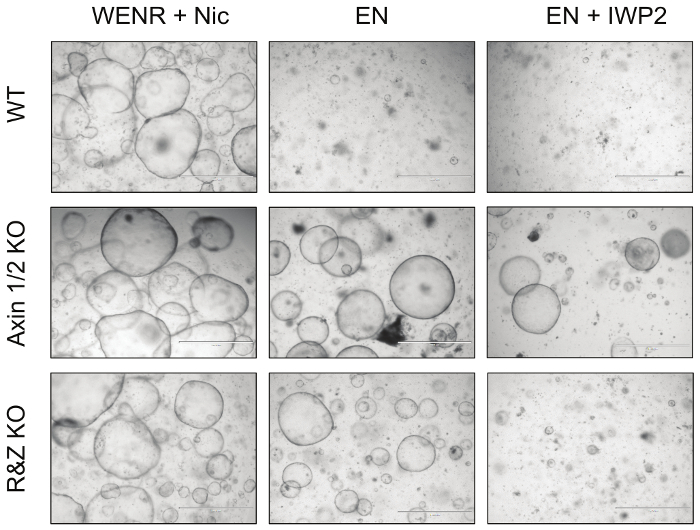

**Figure 4: Representative Images of Mutant Intestinal Organoids. **Knockout of the negative regulators of the Wnt pathway Axin1 and Rnf43, together with their paralogues, renders the intestinal organoids resistant to growth factor deprivation. In particular, Axin1/2 knockout organoids (Axin1/2 KO) can grow in the absence of both R-spondin (EN: EGF + Noggin) and Wnt (EN + IWP2: EN + Porcupine inhibitor), whereas Rnf43/Znrf3 mutant organoids (R&Z KO) can only survive in the absence of R-spondin (EN). In contrast, WT organoids can only survive in the control culture condition, WENR + Nic (Wnt + EGF + Noggin + R-spondin + Nicotinamide). Scale bars = 1,000 µm. Please click here to view a larger version of this figure.

**Table:** 

**Basal medium**		**Comments**
Store at 4 °C for 4 weeks		
Cell culture medium	500 mL	See table of materials
L-Glutamine 100x	5 mL	
Buffering agent 1 M	5 mL	See table of materials
Penicillin Streptomycin 100x	5 mL	
**WENR + Nic (Wnt + EGF + Noggin + R-spondin + Nicotinamide)**		
Store at 4 °C for 2 weeks		
Basal medium	up to 50 mL	
Neuronal cell serum-free supplement (50x)	1 mL	See table of materials
Neuronal cell serum-free supplement (100x)	500 μL	See table of materials
n-Acetylcysteine (500 mM)	125 μL	
mouse EGF (100 μg/mL)	25 μL	
mouse Noggin (100 μg/mL)	50 μL	
R-Spondin conditioned medium	5 mL	
Wnt3a conditioned medium	25 mL	
Nicotinamide (1 M)	250 μL	
**EN + CHIR + Y-27632 (EGF + Noggin + CHIR + Y-27632)**		
Store at 4 °C for 2 weeks		
Basal medium w/o Penicillin Streptomycin	up to 20 mL	
Neuronal cell serum-free supplement (50x)	400 μL	See table of materials
Neuronal cell serum-free supplement (100x)	200 μL	See table of materials
n-Acetylcysteine (500 mM)	50 μL	
mouse EGF (100 μg/mL)	10 μL	
mouse Noggin (100 μg/mL)	20 μL	
Y-27632 (10 μM)	20 μL	
CHIR99021 (8 μM)	10 μL	
**EN (EGF + Noggin)**		
Store at 4 °C for 4 weeks		
Basal medium	up to 50 mL	
Neuronal cell serum-free supplement (50x)	1 mL	See Table of materials
Neuronal cell serum-free supplement (100x)	500 μL	See Table of materials
n-Acetylcysteine (500 mM)	125 μL	
mouse EGF (100 μg/mL)	25 μL	
mouse Noggin (100 μg/mL)	50 μL	

**Table 2: Organoid Media Composition.**

## Discussion

In this protocol, we detail all the steps necessary to generate CRISPR-concatemers and to apply CRISPR-concatemers in mouse intestinal organoids in order to simultaneously knock out multiple genes. As previously noted, this strategy has several advantages, such as its speed, high efficiency and cost-effectiveness.

In order to successfully perform the whole procedure, there are a few critical aspects to consider. First, it is essential that all gRNA oligos are properly annealed and phosphorylated, as they represent the starting material for the *Bbs*I cloning reaction that in itself is very efficient. Secondly, when electroporating organoids, the more cells used per condition, the higher the maximum possible transfection efficiency. In addition, it is also important that after cell dissociation, small cell clusters predominate over single cells.

Nevertheless, it is possible to encounter technical problems when attempting either the cloning or the transfection for the first time; in the case of problems during gRNA cloning, it is recommended to double check the gRNA oligo sequence and, if correct, select additional bacterial colonies for restriction digestion screening. If transfection efficiency and cell viability are low post-electroporation, then it is advisable to repeat the protocol using more cells per condition and reducing the time of cell dissociation to 3 min.

Although the generation of CRISPR-concatemers is relatively cheap and easy, performing larger scale genetic screens in organoids is not, as the scale is limited by the costs associated with organoid culture and by its labor-intensive nature. It is worth mentioning in this case that the CRISPR-concatemer method is also compatible with cell lines, such as HEK293 and mouse embryonic stem cells.

Regardless of the cellular system, another potential drawback of this strategy can be encountered when aiming at the simultaneous knockout of three or four different genes. For instance, each gRNA will have a different targeting efficiency and the changes of hitting all the genes at the same time can be relatively low; for this reason, it is advisable to employ the concatemer system to direct more than one gRNA against the same gene.

Alternative strategies similarly based on Golden Gate shuffling have been proposed over the years to generate multiplex gRNA vectors^7^,^8^. However, in our method it is possible to directly assemble multiple gRNAs into a single retroviral vector in a single round of cloning, which makes it suitable for generating gRNA libraries to target paralogues.

Our CRISPR-concatemer is built in the MSCV retroviral vector backbone. Thus, gRNA concatemer-containing retrovirus can be used to generate stable cell lines that overexpress gRNAs. When combined with a Cas9-inducible system, one can perform inducible paralogue knockouts using our system.

In summary, here we describe how to clone up to four different gRNAs into the same vector in one step and how to apply this strategy to organoid culture with a high transfection efficiency. Furthermore, we provide useful suggestions to maximize the chances of success throughout the entire procedure.

## Disclosures

The authors have nothing to disclose. The authors have no conflict of interest declared.
